# Reinstatement of the Loyalty Islands Sandalwood, Santalum austrocaledonicum var. glabrum (Santalaceae), in New Caledonia

**DOI:** 10.3897/phytokeys.56.5924

**Published:** 2015-10-09

**Authors:** Jean-François Butaud

**Affiliations:** 1 Conservation International, 58 bis avenue de la Victoire, 98800 Nouméa, New Caledonia; 2 Consultant in forestry and Polynesian botany, P.O. Box 52832 - 98716 Pirae, Tahiti, French Polynesia

**Keywords:** *Santalum austrocaledonicum*, Santalaceae, Loyalty Islands, New Caledonia, essential oil

## Abstract

Sandalwoods encompass 19 species restricted to southeast Asia and the Pacific. The species *Santalum austrocaledonicum* Vieill. (Santalaceae) is endemic to New Caledonia (Grande-Terre, Isle of Pines, Loyalty Islands) and Vanuatu, where several varieties are recognized. The Loyalty Islands sandalwood variety is here reinstated as Santalum austrocaledonicum var. glabrum Hürl. emend. Butaud & P.Firmenich, mut. char. It was previously considered a synonym of the type variety; however, new morphological and genetic studies confirmed its distinctiveness. The key for New Caledonian varieties of *Santalum austrocaledonicum* has been updated and a short description of its essential oil composition and organoleptic quality is given.

## Introduction

Sandalwoods are shrubs or small trees well known for the essential oil extracted from their fragrant heartwood and used in perfumery. They belong to the genus *Santalum* (Santalaceae) comprising 19 species restricted to southeast Asia and the Pacific (Harbaugh and Baldwin 2007, Harbaugh 2007, Harbaugh et al. 2010). The most sought-after species is the Indian sandalwood, *Santalum album* L.

*Santalum austrocaledonicum* Vieill. is the only native sandalwood in New Caledonia and Vanuatu (Guillaumin 1925, Hallé 1988, Harbaugh and Baldwin 2007), and is endemic to these archipelagoes. It has been heavily harvested for the past 150 years (Shineberg 1967) and its wood is still exploited for the fragrance industry as its essential oil can be considered as a substitute for Indian sandalwood oil (Braun et al. 2005).

The most recent taxonomical work on *Santalum austrocaledonicum* was carried out by Hallé (1988) who recognized three botanical varieties based on specimens from New Caledonia in Paris (P) herbarium:

- Santalum austrocaledonicum Vieill. var. austrocaledonicum from Grande-Terre (main island of New Caledonia), Isle of Pines, Loyalty Islands and Vanuatu;
- Santalum austrocaledonicum var. pilosulum N.Hallé in the vicinity of Nouméa on the southwest coast of Grande-Terre;
- Santalum austrocaledonicum var. minutum N.Hallé from the northwest coast of Grande-Terre.

Since that revision, several studies have shown the great morphological variations of var. austrocaledonicum in New Caledonia and Vanuatu which are linked to geographical distribution (Quemin 1988, Nasi 1995, Chauvin and Ehrhart 1998, Bottin 2006, Bouvet et al. 2005, Bottin et al. 2007). The main differences in New Caledonia can be summarized as follows:

- Loyalty Islands: large seeds (L = 8–11 mm, D = 7–10 mm), short and wide juvenile leaves (L = 30–52 mm, W = 8–15 mm);
- Isle of Pines: medium-sized seeds (L = 8–10 mm, D = 6–9 mm), long and narrow juvenile leaves (L = 25–76 mm, W = 2–10 mm);
- Grande-Terre: small seeds (L = 6–9 mm, D = 5–7 mm), long and very narrow juvenile leaves (L = 52–70 mm, W = 2–4 mm).

No comprehensive study of the variation of these characters is available in Vanuatu due to lack of herbarium specimens from most of sandalwood populations there.

For this reason, Nasi (1994) intended to describe the Loyalty endemic sandalwood under the variety loyaltensis but his manuscript was never published.

More recently, molecular studies of New Caledonian sandalwood showed strong genetic differentiation between islands and led to the recognition of two evolutionarily significant units, i.e. Grande-Terre and Isle of Pines for the first, and Loyalty Islands for the second (Bottin et al. 2005, 2007).

The New Caledonian sandalwood is subject to exploitation through harvesting and is grown in plantations to satisfy the increasing international demand of sandalwood essential oil. However, its taxonomy appears to be in need of revision to more precisely describe the variability of this New Caledonian biodiversity hotspot species (Myers et al. 2000) and to contribute to the sustainable management of this natural resource.

A first step of this revision is presented here, with the recognition of an endemic sandalwood variety from the Loyalty Islands based on morphological and molecular studies as well as examination of living plants and herbarium specimens.

## Nomenclature

The New Caledonian sandalwood, *Santalum austrocaledonicum*, was described by Vieillard (1861) from samples collected on the hills of Arama, in the Northern extremity of Grande-Terre (*E. Vieillard 1090*, holotype P00645808). Later, it was confirmed that this species was also present on the Isle of Pines, Loyalty Islands and Vanuatu (Guillaumin 1925, 1970, Virot 1950).

In 1964, Hürlimann described a new variety based on a sample collected on the island of Maré in the Loyalty Islands (Stauffer and Hürlimann 1964), Santalum austrocaledonicum var. glabrum Hürl. characterized by glabrous flowers, differing from the type variety (Santalum austrocaledonicum Vieill. var. austrocaledonicum) represented erroneously by samples gathered around Nouméa (Ouen Toro, Anse Vata, Baie de l’Orphelinat) which have villous flowers.

In 1988, the revision of the New Caledonian *Santalum* by Hallé recognized the three varieties mentioned in the introduction. Hallé reduced var. glabrum of Hürlimann to synonymy under the type (autonym) variety, considering it superfluous due to confusion by Hürlimann on what was really the type of the species. Indeed, the latter described var. glabrum in comparison with what is now recognized as var. pilosulum, this one differing morphologically from var. austrocaledonicum. The true var. austrocaledonicum and var. glabrum are in fact identical relative to the morphological differences pointed out by Hürlimann in his diagnosis.

As the native Loyalty Islands sandalwoods are quite homogeneous, only one variety is to be recognized for the entire Loyalty archipelago, which is the one of Hürlimann. Thus, to reinstate the varietal name glabrum for the endemic Loyalty sandalwood, its description must be amended to distinguish it from the true type specimen.

## Systematics

### Santalum austrocaledonicum var. glabrum

Taxon classificationPlantaeSantalalesSantalaceae

Hürl. emend. Butaud & P.Firmenich mut. char.

Santalum austrocaledonicum var. glabrum Hürl., Mém. Mus. Hist. Nat., ser. B, Bot. 15(1): 15 (1964).

#### Type.

New Caledonia, Loyalty Islands, Maré, près de Rawa, arbre, 8 m, en fleurs et en fruits, forêt mésophile, 17 July 1951, *M.G. Baumann-Bodenheim 14762* (holotype: P scan!; isotype: Z scan!).

#### Diagnosis.

Santalum austrocaledonicum var. glabrum is most similar to var. austrocaledonicum in its glabrous inflorescence and leaves wider than 1.5 cm, which differentiates them from the other New Caledonian *Santalum austrocaledonicum* varieties. Santalum austrocaledonicum var. glabrum differs from Santalum austrocaledonicum var. austrocaledonicum by the seed size, which is more than 7.5 mm wide for the former and less than 7.5 mm for the latter.

#### Description.

Shrub to small tree 2–10 m tall, trunk up to 30 cm dbh; bark rough, grey to reddish-brown, longitudinally fissured; heartwood fragrant, yellowish to brownish. ***Leaves*** glabrous; petiole canaliculate, 7–13 mm long; blades of the mature leaves 3.5–6.0 (–6.6) × (1.6–) 2.0–3.5 (–4.1) cm, usually elliptic or rarely obovate, apex obtuse to acute or apiculate, base acute, secondary veins mostly 7–9 pairs. ***Inflorescences*** glabrous, in axillary or terminal panicles, usually trichotomous and several times branched, with 10–40 flowers; peduncles 10–38 mm long. ***Flowers*** bisexual with outer surface of petal greenish and glabrous; pedicels 1.5–2 mm long. ***Petals*** 4, narrowly triangular, 2.5–3.0 × 1.5–2.0 mm, inner surface white when opening turning brownish later; petal internal margin glabrous. ***Stamens*** 4, surrounded by long hairs at the base; the outer ones reaching the anther apex, the inner ones reflexed in the cup-shaped disk; anthers 1.5–2.7 × 0.8–1.0 mm. ***Disk*** concave, more than 2 mm deep; disk lobes fleshy and erected between petals, 1.3–1.4 × 0.8–0.9 mm. ***Ovary*** unilocular, conic, acute, 1.1–1.7 × 0.6 mm; style free, 4 mm long; stigma 3 or 4 lobed. ***Fruit*** a globose fleshy drupe, 15–21 × 12–15 mm when fresh, topped by the petal scars 3–5 mm diameter, green turning red to deep purple and black at maturity. ***Seed*** globose, with a hard endocarp (8.5–) 9.0–11.5 × 7.5–10.0 (–10.5) mm.

#### Phenology.

Flowering and fruiting probably occurring throughout the year but with some peaks; herbarium samples provide the following data: flowers from December to August, fruits from January to August.

#### Distribution.

**Figure 1.**
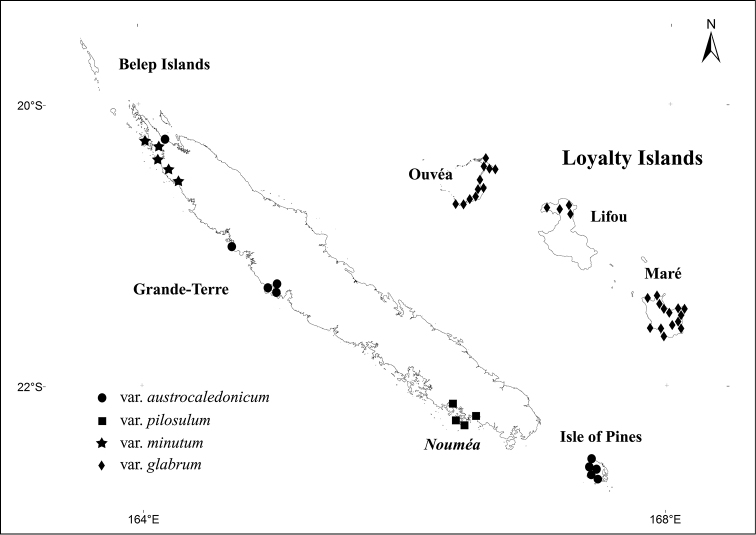
Distribution map of New Caledonian varieties of *Santalum austrocaledonicum* based on the specimens examined.

New Caledonia, endemic to Loyalty Islands; known only from Ouvéa, Lifou and Maré islands (Figure 1). Not recorded on the smaller islands of Beautemps-Beaupré, Tiga and Walpole.

#### Habitat and biology.

This variety is restricted to the calcareous soils of uplifted atolls between 5 and 80 m elevation and is closely linked with traditional agriculture which consists of shifting cultivation. Regeneration by seeds occurs mainly in the open cultivated areas and young fallow lands. Loyalty sandalwood is therefore characteristic of fallows, shrublands and secondary forests, and rarely occurs in mature forests. It is also commonly found along roads and close to villages in open areas where it is generally protected and managed by inhabitants. The surrounding vegetation is often composed of trees and shrubs, such as *Acacia spirorbis* Labill., *Acalypha* spp., *Acronychia laevis* J.R.Forst. & G.Forst., *Dodonaea viscosa* (L.) Jacq., *Elattostachys apetala* (Labill.) Radlk., *Glochidion billardierei* Baill., *Melochia odorata* L.f., *Morinda citrifolia* L., *Pandanus macrocarpus* (Brongn.) Solms, *Podonephelium homei* (Seem.) Radlk., Polyscias bracteata (R.Vig.) Lowry subsp. bracteata., *Psidium guajava* L., and *Schinus terebenthifolius* Raddi. Loyalty sandalwood is a hemiparasitic tree like all *Santalum* species; its pollination is insect-mediated whereas its fleshy fruits are dispersed mainly by doves and pigeons (Bottin et al. 2005).

#### Conservation status.

Using the categories and criteria of IUCN (2001), we propose for Santalum austrocaledonicum var. glabrum the IUCN Red List Category Vulnerable (VU): B (1+2) ab (iii,v). Its population size is estimated at more than 10,000 mature individuals (excluding criteria C and D) with an extent of occurrence around 8,000 km² and an area of occupancy around 1,000 km². Criteria A can not be used due to lack of knowledge of generation length and magnitude of population size reduction. Three locations (one per island) can be distinguished without any fragmentation. A continuing decline is observed and projected in terms of habitat quality and number of mature individuals due to harvest, competition with invasive plant species (*Schinus terebenthifolius* Raddi, *Pluchea odorata* (L.) Cass., *Lantana camara* L.), hybridization with other *Santalum austrocaledonicum* varieties used in plantations, and changes in the traditional agricultural system (less cultivated fields, short fallows...). Indeed, despite provincial regulations establishing quota and exploitability criteria, illegal logging is still occurring (Butaud et al. 2013) whereas hybridization is suspected due to sandalwood interspecific crossability (Tamla et al. 2012).

#### Common names.

The common names recorded for Santalum austrocaledonicum var. glabrum are “tapakae” (pers. obs. 2014) or “tapakai” (Lenormand 1968) on Lifou, “wekesi” (Lormée et al. 2011) on Maré and “wahata” (Ozanne-Rivierre 1984) on Ouvéa.

#### Discussion.

Santalum austrocaledonicum var. glabrum is the sole native sandalwood in the Loyalty Islands. Nevertheless, two other varieties have been introduced for plantation purposes, mainly in Maré and Lifou: var. pilosulum from Ouen Toro in Nouméa, and var. austrocaledonicum from Isle of Pines. These plantations can be considered a risk for the Loyalty variety because of hybridization and subsequent introgression. Plantations with exotic varieties should be discouraged to preserve the Loyalty sandalwood’s morphological, genetic, sylvicultural and chemical specificities (Bottin 2006, Bottin et al. 2007, Butaud et al. 2013, Ehrhart 1998). On the other hand, the sustainable exploitation of natural stands of Loyalty Islands sandalwood is becoming increasingly difficult with the lack of regeneration and the increasing international demand for sandalwood essential oil. Well-managed plantations with variety glabrum could be promoted to preserve the natural stands, to develop the Loyalty sandalwood sector, and to increase the production of heartwood and essential oil (Butaud 2011, Butaud et al. 2013).

The taxonomy of *Santalum austrocaledonicum* still needs to be further investigated. Indeed, the study of herbarium samples of var. austrocaledonicum and previous molecular and morphometric studies (Bottin 2006, Bouvet et al. 2005) showed significant variability. It is expected that future work supported by increased surveys and sampling of northern Grande-Terre sandalwood could reveal one or two new varieties, including one on Isle of Pines (taxon previously described as *Santalum homei* Seem.). Moreover, two new endemic varieties of *Santalum austrocaledonicum* are expected based on the recent study of Millet et al. (2012) on the genetic structure of Vanuatu sandalwood, one for the northern islands and one for the southern islands.

**Figure 2.**
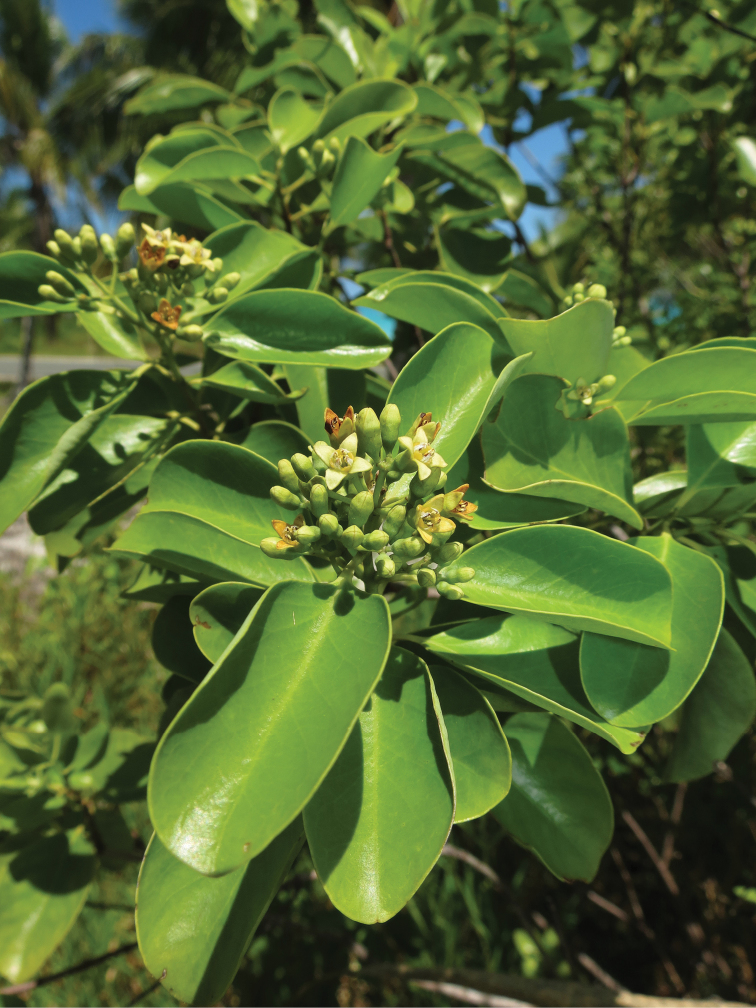
Flowers of Santalum austrocaledonicum var. glabrum on Ouvéa atoll in January 2015 (specimen *Butaud 3414*).

**Figure 3.**
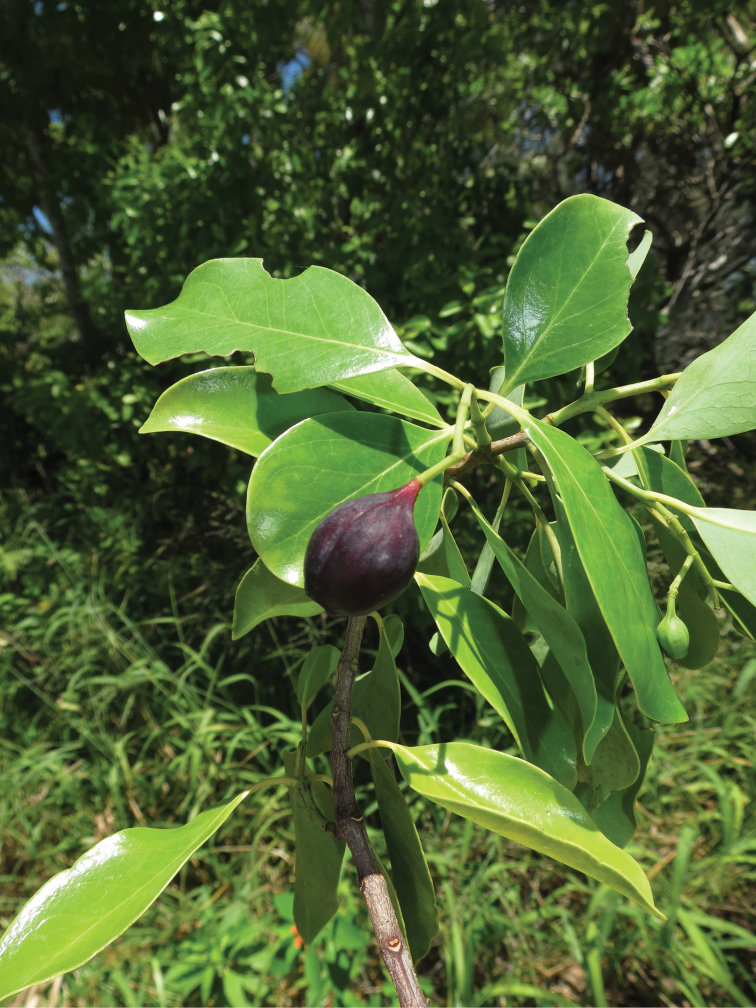
Fruit of Santalum austrocaledonicum var. glabrum on Ouvéa atoll in January 2015 (specimen *Butaud 3414*).

### Key to New Caledonian varieties of *Santalum austrocaledonicum*

(adapted from Hallé 1988)

**Table:** 

1	Inflorescence peduncles, rachis segments and outer surface of petals sparsely to densely hairy	**var. pilosulum**
–	Inflorescence peduncles, rachis segments, outer surface of petals and petal internal margins glabrous	**2**
2	Leaf blade usually less than 3.5 × 1.5 cm; petiole less than 7 mm; blade adaxial surface glaucous and bluish in color; interstaminal disk lobes very narrow (W/L = 1/3–1/4)	**var. minutum**
–	Leaf blade usually more than 3.5 × 1.5 cm; petiole more than 7 mm; blade adaxial surface not glaucous and bluish in color; interstaminal disk lobes wide (W/L = 1/2)	**3**
3	Seed usually more than 9 × 7.5 mm; globose (L/D < 1.2)	**var. glabrum**
–	Seed usually less than 9.5 × 7.5 mm; ovoid (L/D > 1.2)	**var. austrocaledonicum**

### Essential oil quality

Essential oil of *Santalum austrocaledonicum* was previously assessed for its composition and organoleptic properties, and was considered as a possible substitute for Indian sandalwood oil (*Santalum album*) but also as a promising new raw material for the fragrance industry (Braun et al. 2005). These evaluations were performed on essential oils resulting from mixtures of sandalwood varieties glabrum (Loyalty islands), pilosulum (surroundings of Nouméa) and austrocaledonicum (Isle of Pines), leaving unknown the quality of each individual variety.

To investigate the quality of Loyalty Islands sandalwood essential oil (var. glabrum), two samples were analyzed for their main sesquiterpenoids but also for their organoleptic properties:

- Loyalty Oil 1 (EI1) obtained in 2015 from Distillerie de Boulouparis (New Caledonia) with Ouvéa sandalwood,
- Loyalty Oil 2 (EI2) obtained in 2002 from Michel Point and processed by Koop Cuada distillery with Maré and Lifou sandalwoods.

The gas-chromatography was performed on an apolar column (HP-1, 10 m x 0.1 mm, film 0.1m, 50° (1’) to 280° (2’) at 50°/min., vector gas: hydrogen). Its results are given in Table 1 (C. Vial and S.A. Firmenich, pers. comm. 2015).

The organoleptic evaluation showed that the Loyalty essential oil is lighter and less milky than the Indian sandalwood; the former has also a character less woody but more animal and masculine (P.-A. Blanc and S.A. Firmenich, pers. comm. 2015).

**Table 1.** Main constituents of Loyalty Islands sandalwood (var. glabrum) essential oil compared to ISO standards for *Santalum album* oil.

Essential oil	(Z)-α-santalol (%)	(Z)-β-santalol (%)	(E)-lanceol (%)
ISO Standard 3518 : 2002 (*Santalum album*)	41 to 55	16 to 24	-
New Caledonia Braun et al. (2005)	38.2	18.2	9.1
Loyalty Oil 1	44.3	19.4	14.1
Loyalty Oil 2	47.4	21.3	8.5

These findings are in accordance with Braun et al. (2005), especially for the organoleptic evaluation. Moreover, the composition of both Loyalty essential oils meets the ISO Standard for Indian sandalwood, which was not the case for the samples of the previous study. Thus, Santalum austrocaledonicum var. glabrum or Loyalty sandalwood constitutes a true substitute for Indian sandalwood in the perfume industry.

## Supplementary Material

XML Treatment for Santalum austrocaledonicum var. glabrum

## Appendix

**Specimens examined**

Santalum austrocaledonicum var. glabrum Hürl.: New Caledonia, Loyalty Islands.

Ouvéa. Lékine, 15 May 1976, *D. Bourret 815* (NOU); Lekiny, bord de route goudronnée, 24 January 2015, *J.-F. Butaud 3414* (NOU); Lekiny, bord de route goudronnée, 24 January 2015, *J.-F. Butaud 3415* (NOU); Lekiny, bord de route goudronnée, 24 January 2015, *J.-F. Butaud 3416* (NOU); Mouli, bord de route goudronnée, 24 January 2015, *J.-F. Butaud 3417* (NOU); Hnyimehe, route menant à la salle omnisport, 24 January 2015, *J.-F. Butaud 3421* (NOU); Hwaadrila, piste carrossable menant au Cap St Hilaire, 25 January 2015, *J.-F. Butaud 3422* (NOU); St Joseph, 14 August 1925, *A.U. Däniker 2546* (P scan, Z n.v.); Saint-Paul, 10 m, forêt, February 1980, *H.S. MacKee 37848* leg. *Douheret* (NOU, P scan); Nimaha, 29 April 1987, *H.S. MacKee 43516* leg. *Cherrier* (NOU, P scan, Z n.v.); Nimaha, 29 April 1987, *H.S. MacKee 43517* leg. *Cherrier* (P scan); Hanawa, forêt dense littorale, 29 April 1987, *H.S. MacKee 43520* leg. *Cherrier* (P scan); Hanawa, forêt dense littorale, 30 April 1987, *H.S. MacKee 43526* leg. *Cherrier* (NOU, P scan); Pointe de Mouli, 30 April 1987, *H.S. MacKee 43527* leg. *Cherrier* (NOU, P scan); Wakat, 30 April 1987, *H.S. MacKee 43530* leg. Cherrier (NOU, P scan); Wakat, 30 April 1987, *H.S. MacKee 43532* leg. *Cherrier* (NOU, P scan); Gossanat, forêt, 11 May 1987, *H.S. MacKee 43545* leg. *Ongat* (NOU, P scan); Gossanat, forêt, 11 May 1987, *H.S. MacKee 43546* leg. *Ongat* (NOU, P scan); ); Teouta, 11 May 1987, *H.S. MacKee 43547* leg. *Ongat* (NOU, P scan); Teouta, 11 May 1987, *H.S. MacKee 43548* leg. *Ongat* (P scan); Ogniat, en lisière d’une cocoteraie, 11 May 1987, *H.S. MacKee 43549* leg. *Ongat* (P scan); Ogniat, en lisière d’une cocoteraie, 11 May 1987, *H.S. MacKee 43550* leg. *Ongat* (P scan); Takedji, 11 May 1987, *H.S. MacKee 43551* leg. *Ongat* (NOU, P scan); Takedji, 11 May 1987, *H.S. MacKee 43552* leg. *Ongat* (NOU, P scan); 19 July 1984, *P. Morat 7782* (NOU).

Lifou. July 1869, *B. Balansa 1692b* (P scan); 1928, *C. Bergeret 106* (P scan); Mutschaweng, 30 m, lisière de forêt, 18 February 1974, *H.S. MacKee 28169* (P scan, Z n.v.); Natchaom, 5 June 1987, *H.S. MacKee 43577* leg. *Wapae* (P scan); Natchaom, 5 June 1987, *H.S. MacKee 43578* leg. *Wapae* (P scan); Natchaom, 5 June 1987, *H.S. MacKee 43579* leg. *Wapae* (P scan); Ouanaham, 3 August 1987, *H.S. MacKee 43638* leg. Case (NOU, P scan); Hunete, 4 August 1987, *H.S. MacKee 43639* leg. *Case* (P scan); Hunete, dans un jardin, 4 August 1987, *H.S. MacKee 43640* leg. *Case* (NOU, P scan); Hunete, 4 August 1987, *H.S. MacKee 43641* leg. *Case* (NOU, P scan); March 1979, *J.-M. Veillon 3920* leg. *Lespès* (NOU); March 1979, *J.-M. Veillon 3921* leg. *Lespès* (NOU, P scan).

Maré. Medu, dans les fourrés, 22 December 1925, *A.U. Däniker 2546a* (P scan, Z n.v.); *E.I. Franc 1286* (P scan); La Roche, fourré secondaire, 24 April 1987, *H.S. MacKee 43602* leg. *Cornaille* (NOU, P scan); La Roche, fourré secondaire, 24 April 1987, *H.S. MacKee 43603* leg. *Cornaille* (NOU, P scan); Hnadid, 3 August 1987, *H.S. MacKee 43620* leg. *Cornaille* (NOU, P scan); Hnadid, fourré secondaire, 3 August 1987, *H.S. MacKee 43621* leg. *Cornaille* (NOU, P scan); Wakone, fourré secondaire, 3 August 1987, *H.S. MacKee 43622* leg. *Cornaille* (NOU, P scan); Tawainedre, Pewaete, fourré secondaire, 3 August 1987, *H.S. MacKee 43623* leg. *Cornaille* (NOU, P scan); Tawainedre, Pewaete, fourré secondaire, 3 August 1987, *H.S. MacKee 43624* leg. *Cornaille* (NOU, P scan); Cuadene, lisière de forêt, 3 August 1987, *H.S. MacKee 43625* leg. *Cornaille* (NOU, P scan); Penelo, fourré, 3 August 1987, *H.S. MacKee 43626* leg. *Cornaille* (NOU, P scan); Penelo, fourré secondaire, 3 August 1987, *H.S. MacKee 43627* leg. *Cornaille* (NOU, P scan); La Roche, jardin abandonné, 3 August 1987, *H.S. MacKee 43628* leg. *Cornaille* (NOU, P scan); Penelo, Dadac, fourré secondaire, 3 August 1987, *H.S. MacKee 43629* leg. *Cornaille* (NOU, P scan); Peyece, fourré secondaire, 3 August 1987, *H.S. MacKee 43630* leg. *Cornaille* (NOU); Kaewatine, fourré secondaire, 3 August 1987, *H.S. MacKee 43631* leg. *Cornaille* (NOU, P scan); Thogone, fourré secondaire, 3 August 1987, *H.S. MacKee 43632* leg. *Cornaille* (NOU, P scan); Ro, fourré secondaire, 3 August 1987, *H.S. MacKee 43633* leg. *Cornaille* (NOU, P scan); Pede, 3 August 1987, *H.S. MacKee 43634* leg. *Cornaille* (NOU, P scan); Pede, lisière de forêt, 3 August 1987, *H.S. MacKee 43635* leg. *Cornaille* (NOU, P scan); Eni, dans un jardin, 3 August 1987, *H.S. MacKee* 43636 leg. *Cornaille* (NOU, P scan, Z n.v.); Eni, cocoteraie littorale, 3 August 1987, *H.S. MacKee 43637* leg. *Cornaille* (NOU, P scan); 31 August 1987, *H.S. MacKee 43679* (NOU, P scan).

Santalum austrocaledonicum Vieill. var. austrocaledonicum:

New Caledonia, Grande-Terre. Arama, collines près de la mer, 1855-60, *E. Vieillard 1090* (holotype: P scan; isotypes: P [2] scan, K p.p. scan); Arama, collines près de la mer, 1855-60, *E. Vieillard 1090bis* (P scan; K p.p. scan); Vavouto, 26 September 2007, *J. Munzinger 4518* (NOU); Presqu’île de Pindai, propriété Kuhn, 2 July 1987, *J.M. Veillon 6422* (NOU, P scan); Presqu’île de Népoui, Pindai, 100 m, 18 November 1987, *J.M. Veillon 6574* (NOU, P scan); Presqu’île de Pindai, 100 m, 1 April 1988, *J.M. Veillon 6794* (NOU, P scan); Poya, forêt de Nekoro, 5 m, 28 September 1988, *J.M. Veillon 6917* (NOU); Poya, station de Mueo, propriété Johnston entre 10-20 m, 6 May 1998, *J.M. Veillon 8106* (NOU); Poya, station de Mueo, propriété Johnston entre 10-20 m, 6 May 1998, *J.M. Veillon 8107* (NOU, P scan).

New Caledonia, Isle of Pines. 1852, *Home s.n.* (BM scan); 1853, *MacGillivray 818b* (K scan); ); Kuto, 1 February 1980, *H.S. MacKee 37852* leg. *Douheret* (NOU, P scan); Kuto, 9 February 1980, *H.S. MacKee 37853* leg. *Douheret* (NOU, P scan); Kuto, 1 February 1980, *H.S. MacKee 37872* leg. *Sevenet* (NOU, P scan); Kuto, 1 February 1980, *H.S. MacKee 37873* leg. *Sevenet* (NOU, P scan); Plateau, 25 February 1980, *H.S. MacKee 37874* leg. *Sevenet* (NOU, P scan); Ouro, 7 October 1987, *H.S. MacKee 43738* leg. *Quemin* (NOU, P scan); Ouro, 9 October 1987, *H.S. MacKee 43739* leg. *Quemin* (NOU, P scan); Kuto, 9 October 1987, *H.S. MacKee* 43740 leg. *Quemin* (NOU, P scan); Ouameo, 8 October 1987, *H.S. MacKee 43741* leg. *Quemin* (NOU, P scan); Oupotoue, Gadji, 8 October 1987, *H.S. MacKee 43742* leg. *Quemin* (NOU, P scan).

Santalum austrocaledonicum var. pilosulum N.Hallé: New Caledonia, Grande-Terre. Nouméa, Ouen Toro, 50 m, forêt côtière sur pente caillouteuse schisteuse 29 December 1971, *H.S. MacKee 24766* (holotype: P scan); Mont Dore, Ilot Pêcheur, 27 January 2015, *G. Gâteblé 708* (IAC); Paita, Stand de Tir, 27 January 2015, *G. Gâteblé 709* (IAC); Ouen Toro, March-April 1983, *Herbier Convention 10* (NOU); Ouen Toro, 12 February 1979, *M. Hoff 184* (NOU); Nouméa, île Nou, 5 February 1978, *H.S. MacKee 34688* (NOU, P scan); Plantule provenance Ouen Toro, Port-Laguerre, Paita, 1 October 1982, *H.S. MacKee 40837* (NOU, P scan); Nouméa, Ouen Toro, 50 m, 26 June 1987, *H.S. MacKee 43600* (NOU, P scan); Plantule provenance Ouen Toro, Port-Laguerre, Paita, 17 August 1987, *H.S. MacKee 43657* (NOU, P scan); Ouen Toro hill at S End of Noumea, 50 m, 31 January 1982, *G. MacPherson 4558* (NOU, P scan); Nouméa, Anse Vata, SW-Hang des Mt Ouen Toro, 100 m, 22 February 1964, *H.U. Stauffer 5701* (NOU, P scan, Z n.v.); Nouméa, Anse Vata, SW-Hang des Mt Ouen Toro, 100 m, 22 February 1964, *H.U. Stauffer 5702* (NOU, P scan, Z n.v.); Areal der IFO, Anse Vata, Nouméa, 11 March 1964, *H.U. Stauffer 5792* (NOU, P scan, Z n.v.); IFO, Anse Vata, 6 January 1965, *J.M. Veillon 18* (NOU, P scan); Nouméa, Colline du Ouen Toro, 1 April 1969, *J.M. Veillon 1934* (NOU, P scan).

Santalum austrocaledonicum var. minutum N.Hallé: New Caledonia, Grande-Terre. Montagne de Poum, pic 272, versant Est, 50 m, arbuste 3m, 25 March 1982, *J.M. Veillon 4852* (holotype: P n.v.; isotype NOU scan); Arama, collines près de la mer, 1855-60, *E. Vieillard 1090ter* (P scan); Koumac, Camp militaire, SMA, 8 August 1994, *A. Dessert 2* (NOU); Koumac, Babouillat, 14 June 2014, *G. Gâteblé 518* (IAC); Entrée de Koumac, terrain du SMA, 16 December 1994, *T. Jaffré 3257* (NOU, P scan); Au pied de la Tiebaghi 18 October 1970, *M. Schmid 3465* (NOU); Montagne de Poum, 25 March 1982, *B. Suprin 1744* (NOU); Montagne de Poum, 9 June 1982, *B. Suprin 1948* (NOU); Koumac, Camp militaire, SMA, 9 August 1994, *B. Suprin 2435* (NOU).
